# Impact of protein intervention timings on critically ill patients: A systematic review and meta- analysis

**DOI:** 10.2478/jccm-2025-0047

**Published:** 2025-10-31

**Authors:** Pranav Kumar Sharma, Sanjiya Arora, Tirth Bhavsar, Mamta Kamboj, Rahul Kamboj, Varnika Gupta, Anitha Sigamani Ramamurthi, Kumari Uthayakumar, Ajay Singh, Sachin Mahendrakumar Chaudhary, Arghadip Das, Arianisa Bajrami, Sumesh Singh, Devendra Tripathi

**Affiliations:** Department of Internal Medicine, Lake Cumberland Regional Hospital, Somerset, Kentucky, USA; Department of Internal Medicine, Rohilkhand Medical College, Bareilly, Uttar Pradesh, India; Department of Internal Medicine, Smt. NHL Municipal Medical College, Ahmedabad, Gujarat, India; Department of Internal Medicine, Government Medical College, Patiala, Punjab, India; Department of Internal Medicine, King George’s Medical College, Lucknow, Uttar Pradesh, India; Department of Internal Medicine, Lala Lajpat Rai Memorial Medical College,Uttar Pradesh, India; Department of Internal Medicine, PSG Institute of Medical Sciences and Research, Coimbatore, Tamil Nadu, India; Department of Internal medicine, Nanjing Medical University, Nanjing, China; Department of Internal Medicine, Sri Ram Murti Smarak Institute of Medical Sciences, Bareilly, Uttar Pradesh, India; Department of Internal Medicine, Gujarat Cancer Society Medical College, Hospital and Research Center, Ahmedabad, Gujarat, India; Department of Internal Medicine, Nilratan Sircar Medical College and Hospital, Kolkata, West Bengal, India; Department of Internal Medicine, University of Tirana,Albania; Department of Internal Medicine, Institute of Medicine, Tribhuvan University, Kathmandu, Nepal; Department of Internal Medicine, Nazareth Hospital, Philadelphia, PA. USA

**Keywords:** protein supplementation, critical care nutrition, timing of nutritional support, ICU outcomes, meta-analysis

## Abstract

**Background:**

Critically ill patients experience metabolic alterations that promote muscle atrophy and protein catabolism, increasing morbidity and mortality. While adequate protein provision is essential, the optimal timing remains controversial. Guidelines recommend higher protein targets, but evidence from randomized controlled trials is limited and inconsistent.

**Aim:**

To evaluate the effects of early versus late protein supplementation on mortality, complications, and clinical outcomes in critically ill patients.

**Methods:**

A systematic review and meta-analysis were conducted using PubMed, Embase, Cochrane Library, and Google Scholar (January 2010–December 2022). Studies comparing early and late protein administration in adult ICU patients were included. Primary outcomes were mortality, infectious complications, overall complications, pneumonia, ICU/hospital length of stay, and mechanical ventilation duration.

**Results:**

Thirteen studies (8 RCTs, 3 retrospective, 2 prospective cohorts) involving 10,672 patients were analyzed. Mortality (RR = 0.87, 95% CI: 0.74–1.04, p = 0.11; I^2^ = 36%), overall complications (RR = 0.87, 95% CI: 0.74–1.02, p = 0.08; I^2^ = 26%), infectious complications (RR = 0.86, 95% CI: 0.58–1.27, p = 0.37; I^2^ = 65%), and pneumonia (RR = 0.78, 95% CI: 0.41–1.48, p = 0.34; I^2^ = 0%) showed no significant differences between early protein (EP) and late protein (LP) groups. EP significantly reduced ICU length of stay (MD = −0.28 days, 95% CI: −0.33 to −0.23, p < 0.00001; I^2^ = 99%) and mechanical ventilation duration (MD = −0.66 days, 95% CI: −0.90 to −0.41, p < 0.00001; I^2^ = 85%), but was associated with a longer hospital stay (MD = 0.47 days, 95% CI: 0.31–0.63, p < 0.00001; I^2^ = 98%).

**Conclusion:**

Early protein supplementation does not significantly affect mortality or major complications but may shorten ICU stay and ventilation duration. High heterogeneity for some outcomes warrants cautious interpretation.

## Introduction

Patients in critical condition experience metabolic alterations that can have detrimental effects, such as skeletal muscle atrophy and protein catabolism, ultimately leading to increased morbidity and mortality rates [ 1, 2,3]. Survivors may suffer from low muscle mass and persistent weakness [4]. It is well established that timely and adequate protein provision is crucial in the care of critically ill patients [ 5, 6, 7, 8], which has led to the development of current guidelines advocating for higher protein targets than the previously suggested 1.2 g/kg/d [9]. However, the conclusion regarding the optimal protein dosage remains weak due to the limited data from randomized controlled trials. Some studies have shown positive outcomes at doses exceeding the generally recommended range of 1.2–1.5 g/kg/d [ 10,11]. In the context of acute kidney injury, several studies [12,13], though not all [14], have demonstrated efficacy; thus, some experts recommend 2–2.5 g/kg/d in specific patient populations [15]. Nonetheless, uncertainty persists as a recent randomized controlled trial did not confirm this beneficial effect [16].

The optimal timing of protein administration for critically ill patients is a crucial aspect of nutritional prescriptions. Early protein administration in critically ill patients begins within 24–48 hours of intensive care unit (ICU) admission to prevent muscle loss and improve recovery, while late administration occurs after 72 hours and may lead to worse outcomes. Standard protein administration, initiated between 24–72 hours, follows clinical guidelines to optimize nutrition and patient recovery. However, current guidelines do not directly address this issue, and the available data are inconclusive [9, 17]. Casaer et al. suggested in a post hoc analysis that early (day 3) protein supplementation was detrimental to ICU patients’ mortality [18]. It has been proposed to use indirect calorimetry to accurately assess energy requirements when early enteral nutrition is feasible, though it remains unclear if and for whom trophic or hypocaloric goals should be chosen [19]. In this context, targeting lower energy needs complicates the provision of an adequate protein amount. Further research on the optimal timing of protein administration has been called for [20]. To contribute to the existing body of evidence, we conducted a systematic review and meta-analysis of critically ill patients, explicitly examining outcomes in relation to the timing of protein delivery.

## Methods

### Search strategy and eligibility criteria

To identify all relevant studies examining the use of early versus delayed protein administration in critically ill patients, a comprehensive search strategy was employed. Four databases— PubMed, Embase, Cochrane Library, and Google Scholar—were searched, supplemented by hand-searching guidelines, systematic reviews, and reference lists of previous studies. A thorough search strategy was developed using terms such as “early protein,” “delayed protein,” “standard care,” “mortality rates,” “infection rates,” “overall complications,” “length of hospital and ICU stay,” “pneumonia,” “critically ill adults,” and “mechanical ventilation.” Controlled vocabulary techniques, such as Medical Subject Headings (MeSH), were also utilized.

The search focused on English-language articles published between January 2010 and December 2022. The review included clinical trials and prospective and retrospective cohort studies involving patients aged 18 years or older, admitted to an ICU or postoperative unit, who received either early or late protein administration or standard care. The review did not consider the number of calories or protein intake.

The early protein (EP) group served as the experimental group, while the late protein (LP) or standard care groups served as the control groups. Data were collected on the following outcomes: length of hospital and ICU stay, days on mechanical ventilation, mortality rate, infectious complications, overall complications, and pneumonia. All statistics included in the review were derived from the articles identified in the search.

### Selection of studies and data extraction

**Fig. 1.**
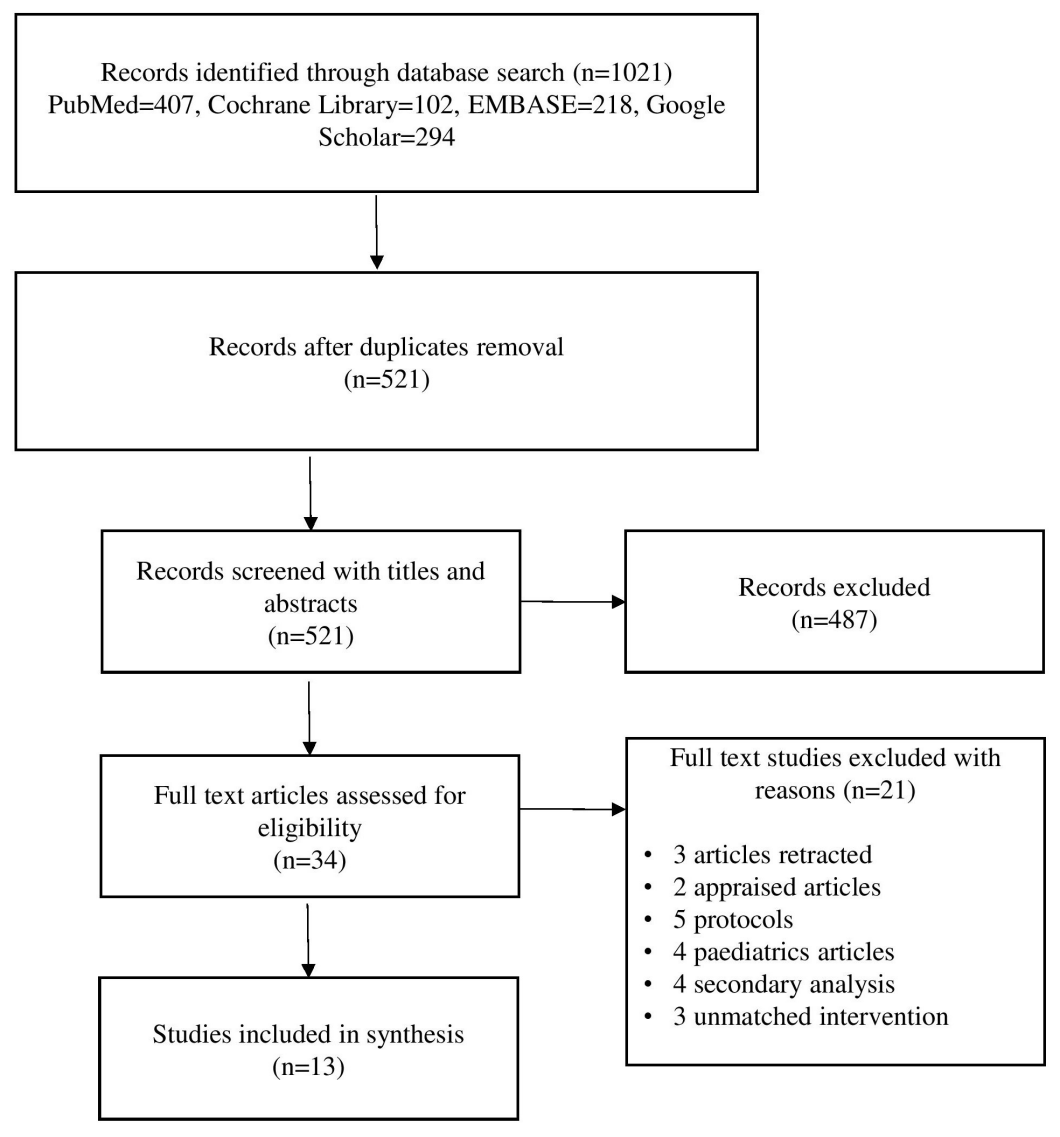
PRISMA flow diagram of search strategy and selection of studies.

Five reviewers independently screened the abstracts and titles to identify potentially relevant studies. These reviewers then retrieved and evaluated the full texts of all studies that met the inclusion criteria. The level of agreement between the two reviewers on the inclusion of studies was assessed, and any disagreements were resolved through discussion and consensus with a third reviewer. The process of identifying and selecting relevant articles is depicted using a PRISMA (Preferred Reporting Items for Systematic Review and Meta-Analysis) flow chart (Figure 1). This analysis included eight randomized controlled trials (RCTs) [ 21, 22, 23, 24, 25, 26, 27, 28], three retrospective [ 29,30,31] and two prospective [ 32, 33] cohort studies with a total of 10672 patients. Seven authors independently extracted data from the selected studies, including relevant outcomes and key characteristics.

### Assessment of risk of bias in the included studies

**Fig. 2.**
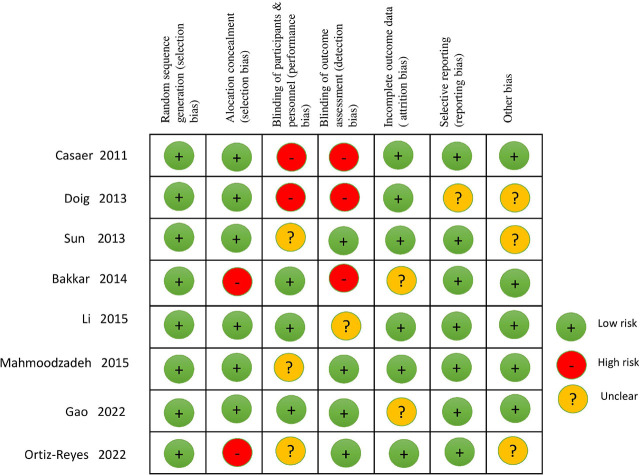
Risk of bias summary: review authors’ judgements about each risk of bias item for each included study

Two authors independently assessed the risk of bias in the included studies using the Cochrane ‘Risk of Bias’ tool [34]. They rated the risk of bias in each domain as low, high, or unclear, resolving any disagreements through discussion, with a third author consulted as necessary. None of the eight included RCTs showed evidence of selection bias (random sequence generation), attrition bias, reporting bias, or other biases [ 21, 22, 23, 24, 25, 26, 27, 28 ]. However, three trials [23, 26,27] exhibited an unclear risk of bias in the area of performance bias. Two trials [24, 28] showed unclear attrition bias, and one trial [25] showed unclear detection bias. Four studies were identified as having a high risk of bias, with two [ 21, 22] showing a high risk of detection and performance bias. Two studies [24, 26] were highly likely to have selection bias due to allocation concealment. Figure 2 provides a graphical summary of the ‘Risk of Bias’ assessments for all included RCTs.

The Newcastle–Ottawa quality assessment scale (NOS) for observational studies [29,30,33] was used to assess the quality of the study in accordance with Cochrane criteria [35]. Three domains made up this scale: result, comparability, and selection. Studies that satisfied the expectations outlined by the scale measures were awarded stars; studies that obtained one or no stars, or stars in multiple categories, were rated as “poor” or “good.” There were no significant departures from this scale. Evaluation criteria were especially designed to align with our population, intervention, comparison, outcome, and study design (PICOS) (Table 1).

**Table 1.** Quality assessment of selected studies using Newcastle-Ottawa Scale (NoS)

**Study**	**Selection**				**Comparability**	**Outcome of interest**			**Overall Quality of study**
	
	**Representativeness of the exposed cohort**	**Selection of non-exposed cohort**	**Ascertainment of exposure**	**Outcome present at start of study**	**Comparability of cohorts**	**Assessment of outcome**	**Length of follow-up**	**Adequacy of follow-up**	
Vicic [2013]	*	*	*	*		*	*	*	Poor
Song [2017]	*	*	*	*	*	*	*	*	Good
Bendavid [2019]	*	*	*	*	**	*	*	*	Good
Koekkoek [2019]	*	*	*	*	**	*	*	*	Good
Sim [2021]	*	*	*	*	**	*	*	*	Good

The review was conducted in accordance with PRISMA guidelines [36].

### Statistical analysis

For the statistical analysis, only the complications that were mentioned in each of the chosen studies were included. Since length of hospital and ICU stay, days on mechanical ventilation, mortality rate, infectious complications, overall complications, and pneumonia, are the most frequently reported and clinically significant complications associated with ICU patients, these rates were used as outcome measures in this meta-analysis. Forest plots were used to display the pooled estimates of the timings of protein administration related problems, and comparisons between early protein and late protein or standard care were carried out. Using the I^2^ and Chi^2^ tests, the heterogeneity among articles was evaluated. When there was evidence of possible heterogeneity (I^2^ > 50% or p value < 0.1 in the Chi^2^ test), the random effects model was applied. Statistical program R (version: 4.3.0) with Rstudio (version: 2023.03.1+446) was used to analyze the data. The meta-analyses were prepared using the meta and meta for programs. P value < 0.05 was used to determine statistical significance in two-sided data.

## Results

A total of 1021 potentially relevant articles were searched from PubMed, Cochrane Library, EMBASE, and Google Scholar databases. The searching and screening of eligible studies is summarized in a PRISMA flow diagram in Figure 1. A total of 10672 patients were enrolled in the 13 studies with 5503 patients in the experimental group or EP group and 5169 in the control group or the LP group.

### Study Characteristics

The investigations incorporated in the analysis were published from 2011 to 2022. The diagnostic categories included mixed [ 21, 22, 29, 30, 33], cancer surgery [ 25, 26,28], acute pancreatitis [ 23, 24], heart dysfunction [27], stomach infection [31], and burns [32]. The time intervals for measuring protein provision ranged from 4 hours to at least 8 days after admission to the ICU. Enteral nutrition was included in four investigations [23,25,27,33], whereas parenteral nutrition was included in four other studies [21, 22,28,31]. Two studies incorporated a blend of enteral and parenteral feeding [29,30]. Two studies administered enteral nutrition to the experimental group and oral nutrition to the control group [24, 32]. A single research administered exclusively oral nourishment to both groups [26]. The characteristics of the studies that were included are displayed in Table 2.

**Table 2.** Basic characteristics of the selected studies

**Study/Year/Country**	**Study Type**	**Patient**	**Early Protein (EP)**	**Late Protein (LP)**	**Outcome measures**	**Reference No.**
Casaer/ 2011/Belgium	Prospective randomized, control, parallel group, multicenter trial.	Critically ill adults in ICU. Total n=4640 EP=2312 LP=2328	Within 48 hours of ICU	≥8 days of ICU admission	Mortality, length of ICU stay, length of hospital stay, any new infection, metabolic complications, organ failure.	
Doig/2013/Australia	Randomized, single blind, multicenter clinical trial	Critically ill (medical, trauma, and surgical) adults in ICU Total n= 1372 EP= 686 Standard care=686	Within 24 hours of admission	Standard care	Mortality (>60 days), ICU length of stay, hospital length of stay, infection, organ failure	
Vicic/2013/Croatia	Prospective multicenter cohort study	Burns patients in ICU Total n=102 EP= 52 LP=50	Within 4 hours of admission in ICU	After first wound dressing	Mortality, pneumonia, sepsis, renal failure, wound infection, multi-organ failure, bacterial and fungal infections.	
Sun/2013/China	Randomized controlled single center trial	Severe acute pancreatitis patients Total n=60 EP=30 LP=30	Within 48 hours after admission	From the 8^th^ day after admission	Mortality, length of ICU stay, pancreatic infection, multiple organ dysfunction, systemic inflammatory response syndrome (SIPS), surgery	
Bakkar/2014/Netherlands	Randomized controlled multicenter trial	Acute pancreatitis patients Total patients n=205 EP=101 LP=104	Within 24 hours after randomization	Standard care 72 hours after presentation	Mortality, infected pancreatic necrosis, pneumonia, bacteremia, mechanical ventilation, organ failure, ICU length of stay.	
Li/2014/China	Randomized controlled single center trial	Cancer patients undergoing gastric cancer surgery Total n=300 EP=150 LP=150	Post-operative day 2	Standard care	Wound infection, urinary tract infection, post-operative fever, vomiting, nausea, bloating	
Mehmoodzadeh/2014/Iran	Randomized controlled single center trial	Patients with esophageal or upper gastrointestinal malignancies undergoing surgical resection Total n= 109 EP= 54 LP=55	First post-operative day (oral)	No oral nutrition until the first bowel sounds returned and there was resolution of ileus.	Pneumonia, ICU length of stay, hospital length of stay, Rehospitalization, peritonitis, esophageal fistula, paralysis of the recurrent laryngeal nerve.	
Song/2017/Korea	Prospective single center cohort study	Critically ill mechanically ventilated ICU patients Total n=210 EP=34 Standard care=176	Within the first 24 hours of ICU admission	Standard care	ICU Mortality, 28 day mortality, hospital mortality, hospital length of stay.	
Bendavid/2019/Israel	Retrospective single center cohort study	Critically ill ICU patients Total n=2253 EP=1040 LP=1213	Within first 3 days of ICU admission	After first 3 days of ICU admission	Mortality (60 days)	
Koekkoek/2019/Netherlands	Retrospective multicenter cohort study	Critically ill patients on mechanical ventilation Total n=455 EP=337 LP=117	<3 days of ICU admission	After 3 days of ICU admission	ICU mortality, hospital mortality, mortality (6 months), days on mechanical ventilation, ICU length of stay, hospital length of stay.	
Sim/2021/Korea	Retrospective cohort study	Emergency abdominal surgery for intra-abdominal infection Total n= 111 EP= 66 LP= 45	Within 48 hours of ICU admission	After 48 hours	Mortality (30 days), in-hospital mortality, infectious complications, pneumonia, ICU length of stay, hospital length of stay.	
Ortiz-Reyes/2022/USA	Randomized controlled multi-national trial	ICU patients with circulatory shock Total n=626 EP=526 LP=100	<48 hours from ICU admission	>48 hours from ICU admission	Mortality (60 days), multiple organ dysfunction syndrome (MODS), sepsis, days on mechanical ventilation, gut ischemia.	
Gao/2022/China	Randomized controlled multicenter trial	Abdominal surgery for gastrointestinal cancers Total n=230 EP=115 LP=115	From day 3 after surgery	From day 8 after surgery	Nasocomial infections, infectious complications, non-infectious complications, adverse events, days on mechanical ventilation, ICU length of stay, hospital length of stay.	

### Mortality outcomes of early versus late protein intervention

**Fig. 3.**
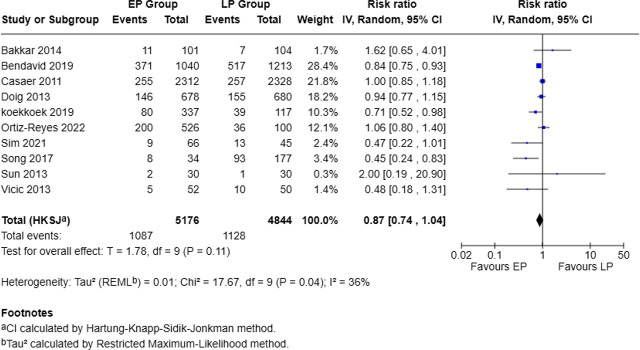
Forest plot for mortality

Mortality was reported in 10 of 13 studies, comprising 5,176 patients in the EP group and 4,844 in the LP group. The crude incidence of death was 21.0% in the EP group versus 23.3% in the LP group. The pooled analysis showed a non-statistically significant reduction in mortality with early protein supplementation (RR = 0.87, 95% CI: 0.74–1.04, p = 0.11). Between-study heterogeneity was low–moderate (Tau^2^ = 0.01; Chi^2^ = 17.67, df = 9, p = 0.04; I^2^ = 36%) (Figure 3).

### Influence of early and late protein on infectious complications

**Fig. 4.**
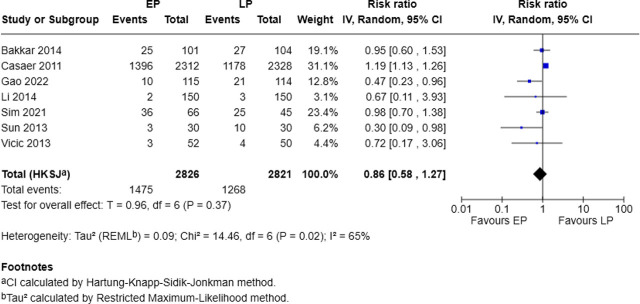
Forest plot for infectious complications

The effect of nutritional intervention timing on infectious complications was investigated in 7 studies with 2,826 patients in the EP group and 2,821 in the LP group. The EP group reported infectious complications in 1,475 patients (52.2%) compared to 1,268 patients (44.9%) in the LP group. The pooled analysis showed a lower, but not statistically significant, risk of infectious complications with early protein supplementation (RR = 0.86, 95% CI: 0.58–1.27, p = 0.37; I^2^ = 65%) (Figure 4).

### Impact of early and late protein on overall complications

**Fig. 5.**
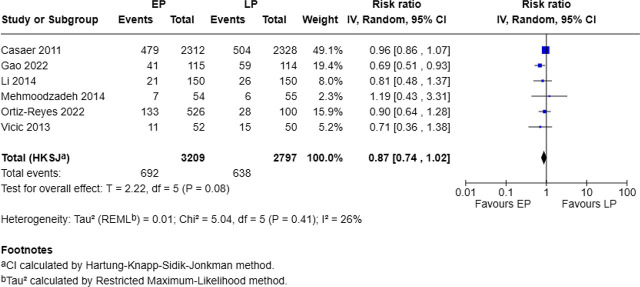
Forest plot for overall complications

Six out of 13 studies reported overall complications, involving 3,209 patients in the EP group and 2,797 in the LP group. The EP group had 692 events (21.5%), while the LP group had 638 events (22.8%). The pooled analysis suggested a non-significant reduction in overall complications with early protein supplementation (RR = 0.87, 95% CI: 0.74–1.02, p = 0.08; I^2^ = 26%), with low heterogeneity (Chi^2^ = 5.04, p = 0.41) (Figure 5).

### Pneumonia associated with timings of protein intervention

**Fig. 6.**
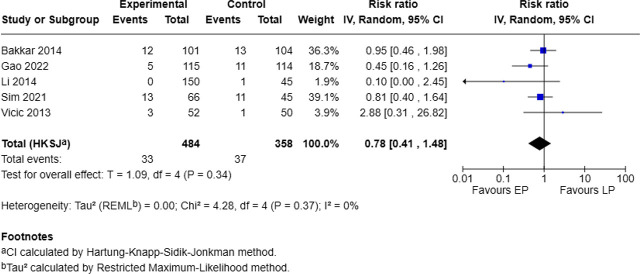
Forest plot for pneumonia

Five studies, involving 484 patients in the EP group and 358 in the LP group, reported pneumonia. The pooled analysis showed no statistically significant difference between groups (RR = 0.78, 95% CI: 0.41–1.48, p = 0.34), with no evidence of heterogeneity (I^2^ = 0%; Chi^2^ = 4.28, p = 0.37) (Figure 6).

### ICU length of stay (ICU LOS) associated with timing of protein support

**Fig. 7.**
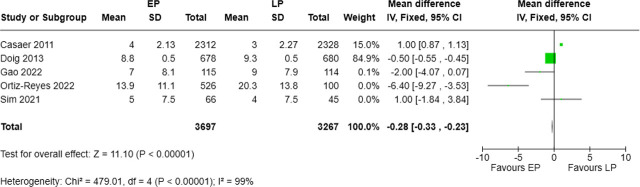
Forest plot for ICU length of stay

Five studies, involving 3,697 patients in the EP group and 3,267 in the LP group, reported ICU length of stay. The pooled analysis showed that early protein supplementation significantly reduced ICU LOS by a mean of 0.28 days (MD = −0.28, 95% CI: −0.33 to −0.23, p < 0.00001). Heterogeneity was very high (I^2^ = 99%; Chi^2^ = 479.01, p < 0.00001) (Figure 7).

### Hospital length of stay (H LOS) associated with timing of protein intervention

**Fig. 8.**
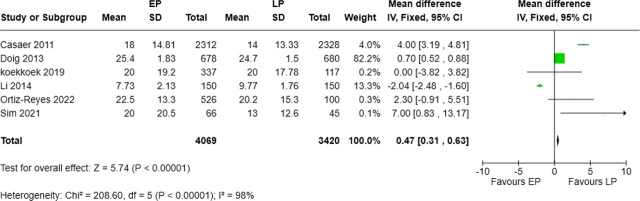
Forest plot for hospital length of stay

Six studies, involving 4,069 patients in the EP group and 3,420 in the LP group, reported hospital length of stay. The pooled analysis indicated that early protein supplementation was associated with a significantly longer hospital stay (MD = 0.47 days, 95% CI: 0.31–0.63, p < 0.00001). Heterogeneity was extremely high (I^2^ = 98%; Chi^2^ = 208.60, p < 0.00001) (Figure 8).

### Comparison of number of days on mechanical ventilation (MV) between the two groups

**Fig. 9.**
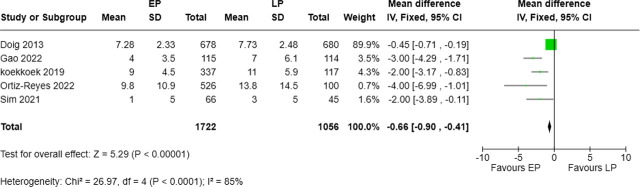
Forest plot for number of days on mechanical ventilation

Five studies, involving 1,722 patients in the EP group and 1,056 in the LP group, reported duration of mechanical ventilation. The pooled analysis showed a significant reduction in ventilation days with early protein supplementation (MD = −0.66 days, 95% CI: −0.90 to −0.41, p < 0.00001). Substantial heterogeneity was present (I^2^ = 85%; Chi^2^ = 26.97, p < 0.0001) (Figure 9).

## Discussion

Enteral or parenteral feedings serve as the primary nutritional source for ICU patients on MV. Numerous studies have investigated nutrition in ICU patients, exploring aspects such as the type, timing, and specific nutrients, with a particular emphasis on protein. The literature presents conflicting results on the effects of early protein supplementation versus late protein supplementation (or usual care) in critically ill patients, specifically concerning variables such as mortality, overall complications, infectious complications, ICU stay, overall hospital stay, and duration of MV. To address this issue, we conducted a systematic review and meta-analysis of 13 studies, including 8 randomized controlled trials, 3 retrospective cohort studies, and 2 prospective cohort studies.

To our knowledge, this meta-analysis on the impact of protein delivery timing in critically ill patients is the largest conducted to date, including 10,672 patients. Our meta-analysis revealed several key findings. Although mortality was numerically lower in the early protein groups, the pooled estimate did not reach statistical significance and there was low–moderate heterogeneity across studies (I^2^ = 36%). Similarly, although the EP groups had a lower rate of infectious complications, the difference was not statistically significant. There were no significant differences between the EP and LP groups concerning overall complications (infectious complications, gastrointestinal issues, metabolic disturbances, organ dysfunction, and prolonged ICU stay) and pneumonia occurrence. Early protein supplementation was associated with statistically significant reductions in ICU length of stay and mechanical ventilation duration, as well as a modest increase in hospital length of stay. However, the substantial heterogeneity for these outcomes (I^2^ = 99% for ICU LOS, I^2^ = 98% for hospital LOS, and I^2^ = 85% for ventilation duration) indicates that these findings should be interpreted with caution, as differences in study populations, intervention protocols, and clinical practices may have influenced the pooled estimates.

In comparison to low and high late protein supplementation, Bendavid et al. found that early protein supplementation in mixed ICU patients was associated with a significantly improved survival rate (HR 0.83, 95% CI 0.71–0.97, p = 0.017) [29]. Similarly, a prospective analysis by Weijs et al. [15] in 2012 involving 843 critically ill mixed patients demonstrated that early protein supplementation enhanced patient survival. Additionally, an observational study by Song et al. [33] indicated that patients who achieved over 90% of their protein target within the first week had a higher likelihood of survival, regardless of whether energy targets were met.

Doig et al. reported no impact on mortality when patients received more energy and protein in the EP group; however, there was improved weaning from mechanical ventilation and reduced muscle mass loss [22]. Koekkoek et al. and Yeh et al., in their retrospective studies, observed that early high protein in-take (within 3–5 days) was linked to worse outcomes [30, 37]. Similarly, Allingstrup et al. found no beneficial effect of early protein supplementation [16]. In a post hoc analysis, Casaer et al. suggested that early administration of protein (by day 3) was detrimental to ICU patients concerning mortality [21]. Koekkoek et al. discovered a time-dependent relationship between protein intake and mortality [30]. Specifically, low protein intake (<0.8 g/kg/day) before day 3 and high protein intake (>0.8 g/kg/day) after day 3 were associated with lower 6-month mortality (adjusted HR 0.609; 95% CI 0.480–0.772, p < 0.001) compared to patients with consistently high protein intake. The lowest 6-month mortality occurred when protein intake increased from <0.8 g/kg/day on days 1–2 to 0.8–1.2 g/kg/day on days 3–5, and then to >1.2 g/kg/day after day 5. Additionally, consistently low protein intake was linked to the highest ICU, in-hospital, and 6-month mortality. The study found no significant differences in ICU length of stay, need for renal replacement therapy, or ventilation duration. It is important to note that in the studies included in this meta-analysis, protein intake in the EP group ranged from 0.4 g/kg/day to over 2.2 g/kg/day, whereas in the LP group, it varied between 0.4 and 1.67 g/kg/day as part of standard care. The wide variation in protein intake across studies may have influenced the results, as higher protein doses could have improved muscle preservation and recovery, whereas lower doses may have limited the potential benefits of early supplementation.

Furthermore, Casaer et al. found that early parenteral nutrition was associated with more frequent infections, longer periods of ventilation and renal replacement therapy, increased cholestasis, and higher hospital costs. Similarly, another RCT involving pediatric population (PEPaNIC)—reported significant harm from early supplementation of inadequate or contraindicated enteral nutrition with parenteral nutrition [38]. Specifically, providing early supplemental parenteral nutrition extended ICU dependency, increased reliance on vital organ support, and raised the incidence of new infections compared to withholding supplemental parenteral nutrition until one week after ICU admission. Among adults, early supplemental parenteral nutrition further increased the incidence of ICU-acquired weakness and hindered recovery [39]. Theoretically, the harm caused by early supplemental parenteral nutrition could be due to the increased nutritional dose or the inferior feeding route. However, two large RCTs in adults—the CALORIES (N = 2400) and Nutrirea-2 (N = 2410) trials—showed no harm from parenteral nutrition when provided in isocaloric doses comparable to enteral nutrition in the early nutritional support [40,41], suggesting that the harm observed in the EPaNIC and PEPaNIC RCTs is likely due to the higher early nutritional dose rather than the intravenous route.

One explanation for better results with enteral nutrition may be attributed to it being more physiologically aligned with gut function, may help maintain gut integrity and reduce the risk of infections, whereas parenteral nutrition, though useful when enteral feeding is not feasible, has been associated with a higher risk of metabolic complications and infections due to factors such as bypassing the gut barrier, increasing susceptibility to bacterial translocation, the risk of catheter-related bloodstream infections, metabolic imbalances like hyperglycemia and electrolyte disturbances, and reduced stimulation of gut-associated immune defenses.

### Strengths and limitations of the study

We considered a diverse range of variables when comparing EP and LP groups, including higher number of patients compared to previous reviews [42,43]. Additionally, Ruijvan et al. did not specify a timeframe in which the amount of protein had to be administered. thereby enhancing the power of our analysis. Our study included eight RCTs, three retrospective, and two prospective cohort studies, encompassing a total of 10,672 patients. The heterogeneity of the various studies included in the meta-analysis, across subgroups of mortality, infectious complications, overall complications, pneumonia, and length of MV, falls within an acceptable range.

The limitations of this meta-analysis encompass several key factors that were not taken into consideration in the studies included. Although we are aware that disease severity and energy provision significantly impact patient outcomes, our meta-analyses were not corrected for these aspects. Unsurprisingly, most studies found that the group with a high protein intake also received more energy, which could result in an underestimating of the positive impact of high protein intake. In addition, this review encompasses investigations, some of which were not specifically meant to investigate certain protein provision categories. Furthermore, we did not consider whether studies made adjustments for protein intake in patients with a body mass index (BMI) of 27.5 or higher. Although the trials did not provide adequate subgroups for subgroup analysis, it is important to consider this because prior research has demonstrated that a universal approach is not appropriate. The ideal protein provision may vary across different patient groups.

### Heterogeneity Considerations

Several outcomes in our meta-analysis, particularly ICU length of stay (I^2^ = 99%) and hospital length of stay (I^2^ = 98%), demonstrated extremely high heterogeneity. This variability is likely multifactorial. First, the included studies differed in patient populations, ranging from medical ICU patients to postoperative surgical ICU cohorts, each with distinct baseline risks and recovery trajectories. Second, definitions and timing of “early” and “late” protein supplementation varied across trials, with protein doses ranging from suboptimal (<1.0 g/kg/day) to near-target levels (>1.5 g/kg/day), which could influence the physiological impact on outcomes. Third, differences in concomitant nutrition strategies, such as total caloric delivery and use of parenteral nutrition, may have contributed to the observed heterogeneity.

Additionally, non-clinical factors such as institutional discharge policies, ICU bed availability, and local weaning practices for mechanical ventilation could have affected length-of-stay metrics. The presence of both randomized controlled trials and observational studies in the pooled analysis further adds methodological variability. Given these factors, results for outcomes with I^2^ > 90% should be interpreted with caution, and future trials should aim for standardized definitions, dosing protocols, and patient selection criteria to improve comparability.

## Conclusion

In this systematic review and meta-analysis, we included 13 studies to evaluate different variables in a variety of patient groups, with a total of 10883 patients. Early protein supplementation in critically ill ICU patients did not demonstrate a statistically significant advantage over late protein supplementation or standard care in terms of mortality, infectious complications, overall complications, or pneumonia. However, the early protein groups did show a statistically significant reduction in ICU LOS and MV duration compared to other groups. The lack of clear benefit from early protein supplementation across various subgroups, including mortality, is attributed to factors such as suppressed autophagy, reduced ketogenesis, and feeding-resistant catabolism.

Despite a decade of RCTs, the optimal timing and dosage of protein supplements remain uncertain. Before recommending such feeding strategies in clinical practice, large-scale RCTs are necessary. These trials should incorporate feeding protocols that preserve beneficial fasting mechanisms like autophagy and ketogenesis without inducing starvation, tailored to different clinical endpoints and patient subgroups. The inclusion of indirect calorimetry and biomarkers will enhance the individualization of feeding strategies for critically ill patients.
